# Activating AMPK to Restore Tight Junction Assembly in Intestinal Epithelium and to Attenuate Experimental Colitis by Metformin

**DOI:** 10.3389/fphar.2018.00761

**Published:** 2018-07-16

**Authors:** Lu Chen, Jie Wang, Qian You, Shuai He, Qianqian Meng, Jian Gao, Xudong Wu, Yan Shen, Yang Sun, Xuefeng Wu, Qiang Xu

**Affiliations:** State Key Laboratory of Pharmaceutical Biotechnology, School of Life Sciences, Nanjing University, Nanjing, China

**Keywords:** AMPK, IBD, tight junction, colonic epithelial cells, metformin

## Abstract

Adenosine monophosphate-activated protein kinase (AMPK), a crucial molecule in energy metabolism, is reported to play a potential role in gut epithelial differentiation and barrier function recently; however, its performance and mechanisms in the pathological process of inflammatory bowel diseases remain unidentified. In this study, we have found that the phosphorylation of AMPK in colonic tissues is negatively correlated with severity of disease during the initiation and development of experimental colitis induced by dextran sulfate sodium. Activation of AMPK by metformin significantly controls the progression of colitis, which is associated with the maintenance of tight junction in colonic epithelium in mice. Moreover, our *in vitro* data in colonic epithelial Caco2 cells shows that metformin promotes expression and assembly of tight junctions via an AMPK-dependent way. Overall, our results suggested that activating AMPK by a clinically safe drug metformin could be a beneficial choice for colitis treatment.

## Introduction

Pain relief, immunosuppression and anti-bacterial have been regarded as main strategies in the treatment of inflammatory bowel diseases (IBD); however, the importance of intestinal barrier maintenance in protection against colitis was somehow underestimated. Intestinal barrier is composed of epithelial cells sealed by tight junctions, which prevent the permeation of pathogens, toxins, and antigens from the luminal environment into the mucosal tissues. Tight junctions, consist of multiple proteins such as transmembrane proteins: occludin, claudins, and junctional adhesion molecules (JAM) and cytoplasmic adaptor proteins such as zonula occludens-1 (ZO-1), are critical in the function of epithelial barrier. During the initiation of IBD, tight junction functions are impaired and substantially precede the development of the disease. Therefore, elucidating the changes of tight junctions and targeting their upstream regulators may be effective for the treatment of IBD.

Adenosine monophosphate–activated kinase (AMPK) is an important energy sensor regulating energy homeostasis and metabolic stress in many cells. The inactivation condition of AMPK contributes to many pathologic processes, such as diabetes, tumor, aging, and inflammatory diseases (Burkewitz et al., 2014; Burkle and Hanfling, 2015). AMPK phosphorylation is reported to be involved in tight junction assembly and cell polarization in Madin-Darby canine kidney (MDCK) cells (Zhang et al., 2006; Zheng and Cantley, 2007), but still remains elusive in many other cells. Recently, it is shown that AMPK enhances intestinal barrier function and epithelial differentiation via promoting CDX2 expression (Silberg et al., 2000). However, characteristics and functional implications of AMPK in the process of IBD are under investigated in colonic epithelium, an essential and the first station for processing inflammatory information. We examined alterations of phosphorylation for AMPK subunits in the colons after inflammation induction and further examined whether and how administration of metformin, the most widely prescribed oral AMPK activator, might restore tight junction function and inhibit dextran sulfate sodium (DSS)-induced experimental colitis.

## Materials and Methods

### Animals

Six to eight-week male C57BL/6 mice were obtained from the Model Animal Genetics Research Center of Nanjing University (Nanjing, China). Animal welfare and experimental procedures were carried out strictly in accordance with the recommendation of Guide for the Care and Use of Laboratory Animals (Ministry of Science and Technology of China, 2006), and the Nanjing University Animal Care and Use Committee (NJU-ACUC). The protocol was approved by the Nanjing University Animal Care and Use Committee (NJU-ACUC) and to minimize suffering and to reduce the number of mice used.

### Reagents

Dextran sulfate sodium was purchased from MP Biomedicals (Aurora, OH, United States). Compound C and AICAR were purchased from MedChemExpress (MCE, United States). Antibody for phosphorylated AMPK (Thr172) was purchased from Cell Signaling Technology (Beverly, MA, United States), antibodies for AMPKα1/2, ZO-1, occludin, claudin-1, Actin, Tubulin were purchased from Santa Cruz (Santa Cruz, CA, United States), IgG for rabbit and mouse were purchased from (Beyotime Biotechnology, China). Human recombinant interferon-γ (IFN-γ) was purchased from R&D Systems (Minneapolis, MN, United States). Lipopolysaccharide (LPS), metformin, mesalazine and other chemicals were purchased from Sigma-Aldrich (St. Louis, MO, United States). Alexa Fluor 488-conjugated anti-rabbit IgG was purchased from Life Technology (Carlsbad, CA, United States)

### Induction and Treatment of Colitis

2.5% (wt/vol) DSS was utilized to induce acute colitis in mice. Mice administrated with DSS dissolved in drinking water continuously from day 0 to day 7. Normal mice received the same drinking water without DSS. Metformin, mesalazine and AICAR were dissolved in normal saline, Compound C was diluted in normal saline from stock solution, which was dissolved in dimethyl sulfoxide (DMSO). Metformin (125, 250, 500 mg/kg, *p.o.*), mesalazine (200 mg/kg, *p.o.*), AICAR (100 mg/kg, *i.p.*) and Compound C (10 mg/kg, *i.p.*) were administered once a day from day 0 to day 11 (*n* = 6–8 mice per group). Weight, morbidity and the presence of gross blood in feces and at the anus were observed daily. The disease activity index (DAI) was calculated by assigning well-established and validated scores as previously described (Wu et al., 2014). That is, the following parameters were used for calculation: (a) diarrhea (0 points = normal, 2 points = loose stools, 4 points = watery diarrhea); (b) hematochezia (0 points = no bleeding, 2 points = slight bleeding, 4 points = gross bleeding). At day 9 following induction with DSS, the animals were sacrificed, the entire colon was quickly removed for *ex vivo* study. Segments of the colon taken for histopathological essay were fixed in 10% normal buffered formalin, embedded in paraffin. Sections were stained with hematoxylin and eosin and histological score was evaluated (blinded) as follows: 0, no signs of inflammation; 1, low leukocyte infiltration; 2, moderate leukocyte infiltration; 3, high leukocyte infiltration, moderate fibrosis, high vascular density thickening of the colon wall, moderate goblet cell loss, and focal loss of crypts; and 4, transmural infiltrations, massive loss of goblet cell, extensive fibrosis, and diffuse loss of crypts.

### Isolation of Colonic Epithelial Cells

Mice were sacrificed after anesthesia and a portion of the colons close to the rectum were immediately removed. Epithelial cells were obtained from freshly isolated colons and flushed from both ends with sterile phosphate buffer (PBS). Colons were then opened longitudinally and thoroughly washed in cold PBS. The colon was then incubated in 10 ml 30 mM ethylene diamine tetraacetic acid (EDTA) and 1.5 mM dithiothretol (DTT) on ice for 20 min and then removed and washed in cold PBS and incubated in 10 ml PBS containing 30 mM EDTA at 37°C at 200 RPM for 10 min. The cells were shaking vigorously for 30 s and centrifuged at 1000 *g* for 5 min at 4°C, washed in PBS containing 10% FBS and spun for a further 5 min at 4°C at 1000 *g*. Cell pellets constituted isolated colonic epithelial cell fractions.

### Western Blot

Samples were collected and lysed in a lysis buffer containing protease and phosphatase inhibitors (Pierce). The proteins were fractionated by SDS-PAGE and electrophoretically transferred onto polyvinylidene fluoride membranes. The membrane was blocked with 5% BSA for 2 h at room temperature. Different antibodies were incubated overnight at 4°C. After washing with PBST for 3 times, 10 min/time, membranes were incubated with secondary antibody for 2 h at room temperature. Finally, membranes were washed by PBST for 3 times, 10 min/time, and the software Quantity One (Bio-Rad Laboratories, Hercules, CA, United States) was used for densitometric analysis.

### Real-Time Quantitative PCR

Real-time PCR was performed as described previously (Schmittgen and Livak, 2008). Total RNA of colon tissue was reverse transcribed to cDNA and subjected to quantitative PCR, which was performed with the BioRad CFX96 TouchTM Real-Time PCR Detection System (BioRad, CA, United States) using iQTM SYBR^®^ Green Supermix (BioRad, CA, United States), and threshold cycle numbers were obtained using BioRad CFX Manager software. The program for amplification was 1 cycle of 95°C for 2 min followed by 40 cycles of 95°C for 10 s, 60°C for 30 s, and 95°C for 10 s. The relative gene expression was the comparative C_T_ method. The primer sequences used in this study were as follows:

IL-18 forward 5′-GACTCTTGCGTCAACTTCAAGG-3′; IL-18 reverse 5′-CAGGCTGTCTTTTGTCAACGA-3′; IL-1β forward 5′-CTTCAGGCAGGCAGTATCACTC-3′; IL-1β rev-erse 5′-TGCAGTTGTCTAATGGGAACGT-3′; IL-6 forward 5′-ACAACCACGGCCTTCCCTAC-3′; IL-6 reverse 5′-TCTCATTTCCACGATTTCCCAG-3′; ICAM-1 forward 5′-CTGGCGTAGATCGACTGTGC-3′; ICAM-1 reverse 5′-AGACTCCTTGCTCATGTCAATG-3′; COX-2 forward 5′-TTCAACACACTCTATCACTGGC-3′; COX-2 reverse 5′-AGAAGCGTTTGCGGTACTCAT-3′; iNOS forward 5′-GTTCTCAGCCCAACAATACAAGA-3′; iNOS reverse 5′-GTGGACGGGTCGATGTCAC-3′; β-actin forward 5′-TGCTGTCCCTGTATGCCTCT-3′; β-actin reverse 5′-TTTGATGTCACGCACGATTT-3′.

### Immunofluorescence Histochemistry

For paraffin-embedded colonic tissue, the sections (4–5 μm) were deparaffinized, rehydrated and washed in 1% PBS-Tween 20 (PBST). Then they were blocked with 3% bovine serum albumin (BSA) and incubated for 2 h at room temperature, and incubated with anti-phosphorylated AMPK antibody (1: 100) and IgG (1:100) overnight at 4 C. After washing with PBST for 3 times, 10 min/time, slides were incubated with fluorescein isothiocyanate (FITC)-conjugated anti-rabbit IgG (1:200, at room temperature for 2 h and then stained with 1 μg/mL DAPI (4′,6-diamidino-2-phenylin- dole) for 1 min. Likewise, for Caco2 cells, after treated with 2.5% DSS for 48 h with or without metformin (30 μM), cells were fixed with ice-cold methanol and were permeabilized with 0.3% Triton X-100/PBST. After blocking with 3% BSA for 2 h, the Caco2 cells were incubated with the ZO-1, claudin-1 and occludin antibody (1:100) overnight at 4°C. After washing with PBST for 3 times, 10 min/time, cells were exposed to the FITC-conjugated secondary antibody (1:200, at room temperature for 2 h). Finally, the coverslips were stained with DAPI for 1 min and washed with PBST for 3 times, 10 min/time. Confocal microscopy analyses were carried out using Olympus FV1000 confocal system (Olympus, Japan).

### Immunohistochemical Analysis

Immunohistochemical analysis was performed on paraffin-embedded colon tissue sections (4–5 μm) to evaluate the expression of ZO-1, claudin-1 and occludin. Slides were deparaffinized, rehydrated and blocked as described above, and incubated with ZO-1, claudin-1 and occludin antibody (1:100) overnight at 4°C. After washing with PBST for 3 time, 10 min/time, slides were incubated with streptavidin-HRP (Shanghai Gene Company, GK500705, Shanghai, China) for 40 min, then stained with DAB (Shanghai Gene Company, GK500705, Shanghai, China) substrate for 2–10 min and counter-stained with hematoxylin.

### Cell Culture

Caco2 cells were purchased from Shanghai Institute of Cell Biology (Shanghai, China) and maintained in DMEM medium, supplemented with 100 U/ml of penicillin, 100 μg/ml of streptomycin and 10% fetal calf serum under a humidified 5% (v/v) CO_2_ atmosphere at 37°C.

### Statistical Analysis

Results were expressed as mean ± SEM. of three independent experiments and each experiment included triplicate sets. Data were statistically evaluated by one-way ANOVA followed by Dunnett’s test between control group and multiple dose groups. The level of significance was set at a *P*-value of 0.05.

## Results

### The Phosphorylation Level of AMPK Was Downregulated During the Progression of Colitis Induced by DSS in Mice

**FIGURE 1:**
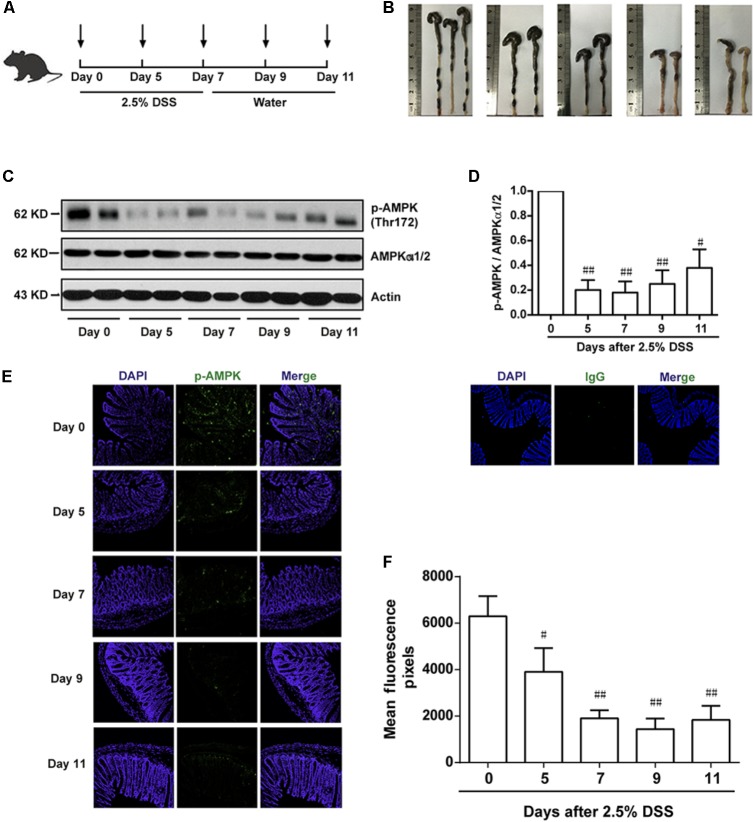
The phosphorylation level of AMPK was downregulated during the progression of colitis induced by DSS in mice. **(A)** Schematic overview of experimental colitis model. **(B)** The length of colon from experimental colitis mice induced by 2.5% DSS at indicated time. **(C,D)** Epithelial cells were isolated from colonic tissue. The phosphorylation level of AMPK (Thr172) and protein level of total AMPKα1/2 were determined by western blot. **(E,F)** 2.5% DSS was used to mimic colitis, sections of colonic tissue were stained for p-AMPK (green), IgG and DAPI (blue) at indicated time. Magnification: 200×,.^#^*P* < 0.05, ^##^*P* < 0.01 *vs.* normal water-treated group.

To investigate the potential role of AMPK during the progression of colitis, the activation of AMPK, which is characterized as phosphorylation on Thr172 in α subunit, was evaluated during the development of colitis induced by DSS. As shown in **Figure 1A**, mice were administrated with 2.5% DSS for 7 consecutive days, and then substituted with water for another 4 days. Colon length was substantially reduced during the progression of colitis, indicating the worsened condition of inflammation (**Figure 1B**). Meanwhile, epithelium was isolated and collected from colons in mice after DSS administration on Day 0, 5, 7 and water administration on Day 9, 11, respectively. It was shown that the activation of AMPK was gradually decreased during DSS administration and then slightly increased after drinking water (**Figures 1C,D**). Consistent with this result, the immunofluorescence of phosphorylated AMPK showed the same tendency (**Figures 1E,F**).

### Metformin Up-Regulated AMPK Phosphorylation and Ameliorated Experimental Colitis Induced by DSS in Mice

**FIGURE 2:**
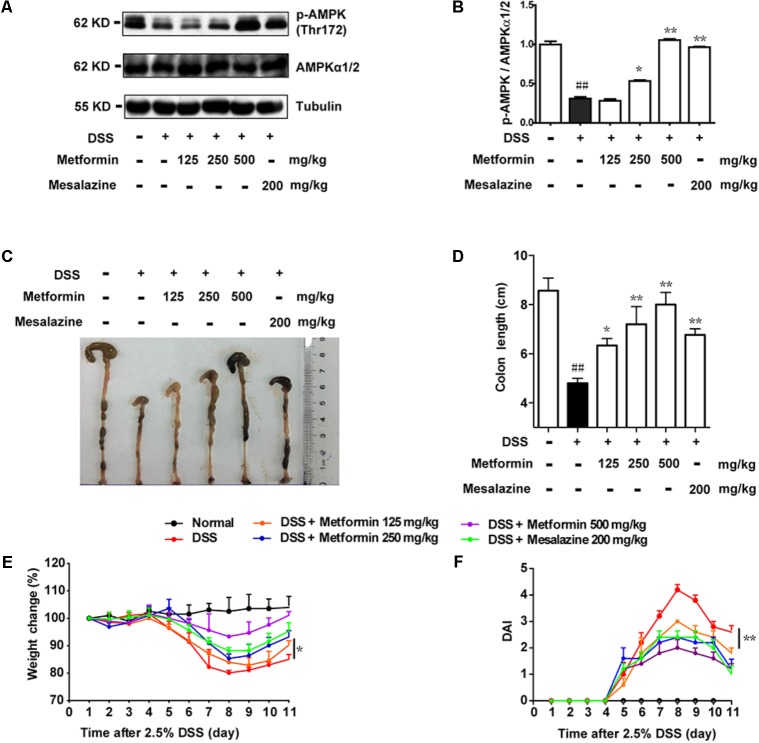
Metformin up-regulated the activation of AMPK and ameliorated experimental colitis induced by DSS in mice. **(A,B)** Epithelial cells from colon tissue were extracted from mice and protein level of p-AMPK (Thr172) and AMPKα1/2 were measured by western blot. **(C,D)** Macroscopic images and the length of colon from each group were measured. Data are presented as means ± SEM (*n* = 6–8 per group). **(E)** Body weight changes for each mouse were measured daily during disease process. **(F)** Disease activity index (DAI) was calculated daily through experiments. ^##^*P* < 0.01 *vs.* normal group, ^∗^*P* < 0.05, ^∗∗^*P* < 0.01 *vs.* DSS-treated alone group.

According to the results in **Figure 1**, we found an inverse correlation between AMPK phosphorylation level and colitis severity. Therefore, we wonder whether promoting AMPK activation could prevent the development of colitis. Since metformin has previously been demonstrated to active AMPK and is considered as a safe drug in clinical application, we use metformin to further study the role of AMPK in colitis. Mesalazine, a cyclooxygenase (COX) inhibitor, is widely used for IBD treatment (Frieri et al., 2017). In the present study, we used mesalazine (200 mg/kg) as a positive control. Mice were divided into 6 groups (*n* = 6–8 per group): normal, DSS alone, DSS with metformin (125, 250, and 500 mg/kg, *p.o.*) and DSS with mesalazine (200 mg/kg, *p.o.*). Colonic epithelial cells were isolated for western blot. As shown in **Figures 2A,B**, compared with DSS group, metformin (125, 250, and 500 mg/kg) exhibited significant effect on AMPK activation. Simultaneously, compared with normal group, mice administrated with 2.5% DSS showed typical symptoms of colitis, characterized as significant weight loss, shortened colon length, and elevated disease activity index (DAI) which reflected severity of colitis (including diarrhea, intestinal bleeding). In contrast, these responses in mice treated with metformin (125, 250, and 500 mg/kg) were attenuated markedly in a dose-dependent manner. In addition, mesalazine showed weaker effects than metformin (500 mg/kg) (**Figures 2C–F**).

### Metformin Attenuated Pathological Damages Induced by DSS in Mice

**FIGURE 3:**
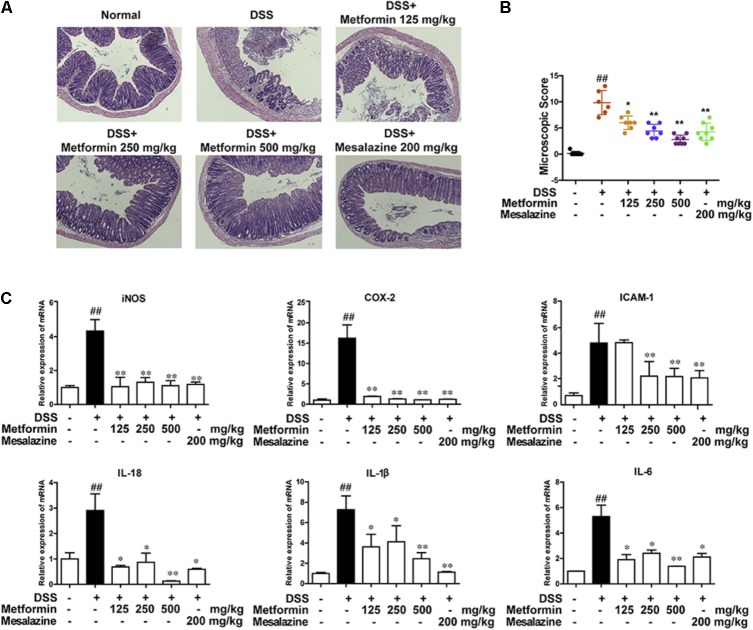
Metformin treatment prevented pathological damage induced by DSS in mice. **(A)** Serial sections of colon tissues were stained with H&E. **(B)** Histopathological scores of each group were determined (*n* = 6–8 per group). **(C)** The mRNA expression iNOS, COX-2, ICAM1, and pro-inflammatory cytokines IL-18, IL-1β, IL-6 in colonic tissues was determined by real-time PCR, and the expression levels were normalized to β-actin (*n* = 6–8 per group). ^##^*P* < 0.01 *vs.* normal group, ^∗^*P* < 0.05, ^∗∗^*P* < 0.01 *vs.* DSS-treated alone group.

To further confirm the inhibitory effect of metformin on colitis, histological changes and cytokine profiles were examined. As shown in **Figures 3A,B**, sections from colon tissue stained with hematoxylin and eosin (H&E) showed a loss of architecture and immune cell infiltration. However, the inflammatory responses were obviously reduced following metformin (125, 250, and 500 mg/kg) or mesalazine (200 mg/kg) treatment. The blinded histological score also revealed that oral administration of metformin was associated with a significant down-regulation in the severity of colitis. Furthermore, inflammation-associated factors such as interleukin-18 (IL-18), interleukin-1β (IL-1β), interleukin-6 (IL-6), Cyclooxygenase2 (COX2), intercellular cell adhesion molecule-1 (ICAM1), and inducible nitric oxide synthase (iNOS) were examined in mRNA level. As showed in **Figure 3C**, IL-18, IL-1β, IL-6, COX2, ICAM1, iNOS were upregulated in DSS-induced inflammation. By contrast, mRNA levels of these factors were significantly decreased in mice treated with metformin or mesalazine.

### Metformin Prevented the Loss of Tight Junction Induced by DSS in Mice

**FIGURE 4:**
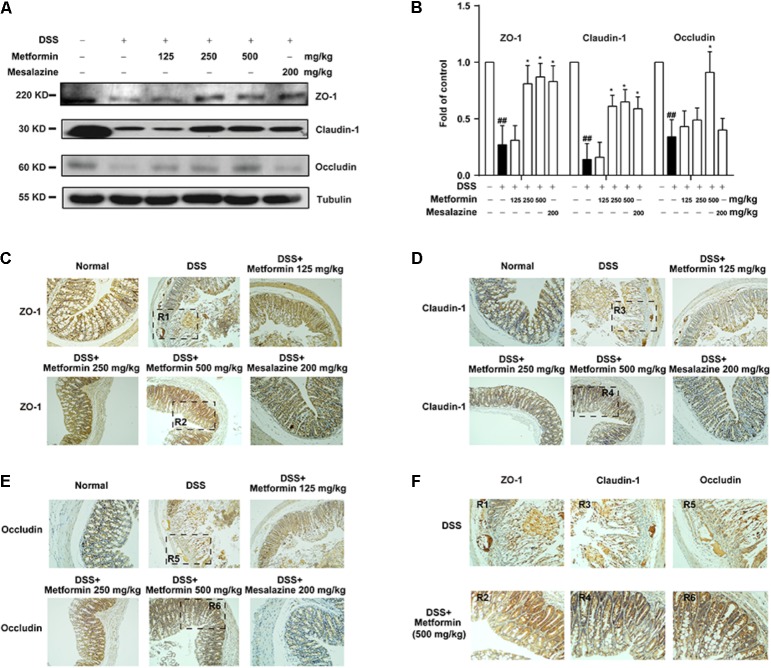
Metformin prevented the loss of tight junction induced by DSS administration in mice. **(A,B)** Intestinal epithelial cells were isolated from normal, DSS, metformin and mesalazine-treated mice at day 11 (*n* = 6–8 per group). The expression of tight junction (ZO-1, claudin-1 and occludin) were examined by western blot. **(C–E)** Paraffin-embedded colon tissue sections from each group were stained for ZO-1, claudin-1 and occludin. **(F)** Magnification of histopathological appearances in some regions (R1-6) of colon wall from DSS (R1, R3, R5) or mice treated with metformin (500 mg/kg) (R2, R4, R6). ^##^*P* < 0.01 *vs.* normal group, ^∗^*P* < 0.05, *vs.* DSS-treated alone group.

To further investigate the effect of metformin on tight junctions (i.e., ZO-1, claudin-1 and occluding proteins) during experimental colitis, colonic epithelium of mice from each group was isolated and the expression of tight junction proteins were examined by western blot and immunohistochemistry. As shown in **Figures 4A,B**, there was a dramatic loss of tight junction proteins staining in mice treated with DSS. Interestingly, concomitant administration of metformin (125, 250, and 500 mg/kg) significantly prevented the loss of tight junction proteins. The immunohistochemistrical results also showed that substantial decrease of these three tight junction proteins was regained in mice treated with metformin or mesalazine (**Figures 4C–F** and Supplementary Figure S1).

### The Effect of Metformin on DSS-Induced Experimental Colitis Was Dependent on AMPK Activation

**FIGURE 5:**
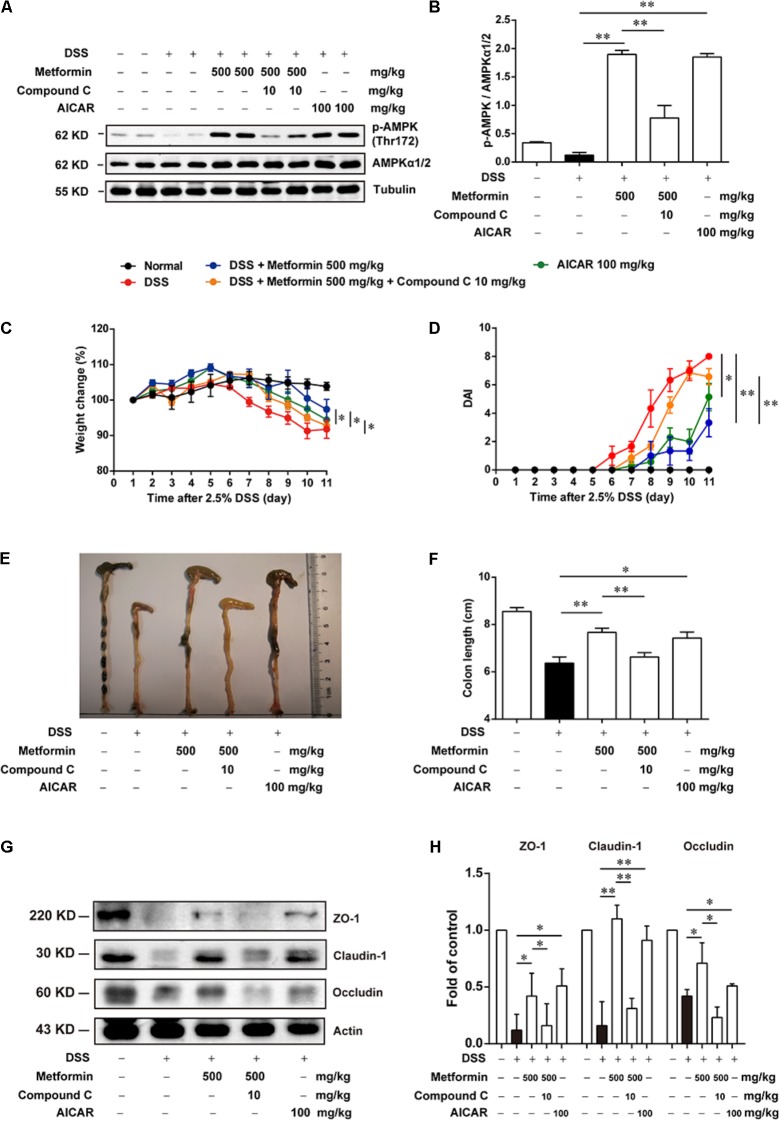
The effect of metformin on DSS-induced experimental colitis was dependent on AMPK activation. **(A,B)** Epithelial cells from colon tissue were extracted from mice in each group (*n* = 6–8 per group) and protein level of p-AMPK (Thr172) and AMPKα1/2 were measured by western blot. **(C)** Body weight changes for each mouse were measured daily during disease process. **(D)** Disease activity index (DAI) was calculated daily through experiments. **(E,F)** Macroscopic images and the length of colon from each group were measured. **(G,H)** Tight junction proteins (i.e., ZO-1, claudin-1 and occludin) from epithelial cells were examined by western blot. Data are presented as means ± SEM. ^∗^*P* < 0.05, ^∗∗^*P* < 0.01.

Since metformin has been reported to have some other targets (Kim et al., 2016), we further used Compound C, a specific inhibitor of AMPK and another AMPK activator, AICAR, to examine whether metformin functioned via AMPK activation. Mice were divided into 5 groups (*n* = 6–8 per group): normal, DSS with vehicle, DSS with metformin (500 mg/kg, *p.o.*), DSS with metformin (500 mg/kg, *p.o.*) and Compound C (10 mg/kg, *i.p.*), DSS with AICAR (100 mg/kg, *i.p.*). Intestinal epithelial cells were isolated from colon tissue for western blot tests (**Figures 5A,B**), we found that both metformin and AICAR activated AMPK significantly, while Compound C could reverse AMPK activation induced by metformin. Similarly, AICAR could also attenuate DSS-induced colitis and promoted the expression of tight junctions. However, the therapeutic effect of metformin on experimental colitis and damaged integrity of intestinal epithelium induced by DSS was almost reversed by Compound C. These data suggested that regulation of tight junctions by metformin occurs in an AMPK-dependent manner (**Figures 5C–H**).

### Metformin Increased the Expression of Tight Junctions in an AMPK-Dependent Way in Caco2 Cells *in Vitro*

**FIGURE 6:**
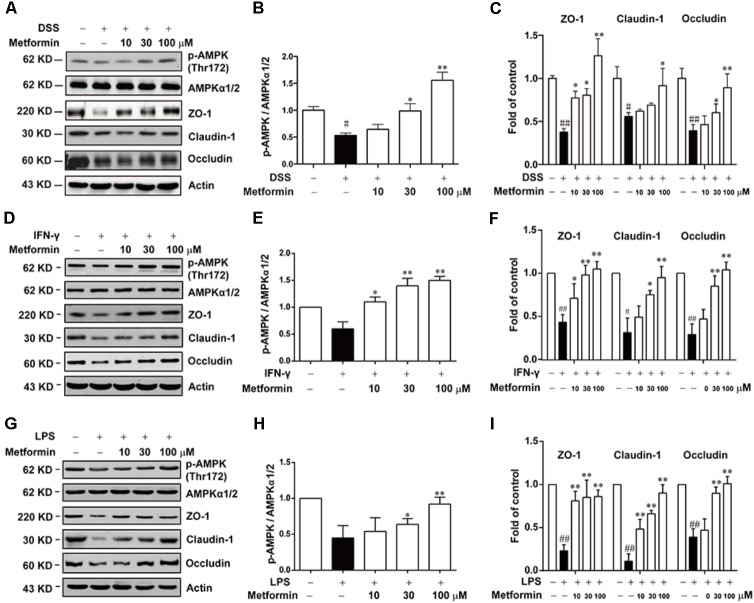
Metformin up-regulated the loss expression of tight junctions induced by DSS, IFN-γ or LPS in Caco2 cells. **(A–C)** Caco2 cells were treated with 2.5% DSS in the presence or absence of metformin (10, 30, and 100 μM) for 48 h. **(D–I)** Caco2 cells were incubated with IFN-γ (1000 U) or LPS (500 ng/ml) in the presence or absence of metformin (10, 30, and 100 μM) for 72 h respectively. The expression of p-AMPK, AMPKα1/2 and tight junction proteins were determined by western blot. Quantifications of immunoreactive signals were performed after normalization to total protein content of each lane. Data are presented as mean ± SEM; ^#^*P* < 0.05, ^##^*P* < 0.01 *vs.* normal group, ^∗^*P* < 0.05, ^∗∗^*P* < 0.01 *vs.* DSS, IFN-γ or LPS-treated alone group.

To further study the mechanism underlying the effect of metformin on tight junction proteins, we used Caco2 cells which could form tight junctions when grew into monolayer. To mimic the circumstance of epithelial cells during colitis, 2.5% DSS, IFN-γ (1000 U) or LPS (500 ng/ml) were applied to mimic epithelial cell damage in colitis *in vitro* respectively (Araki et al., 2006; Cao et al., 2013; Satyam et al., 2017). As shown in **Figures 6A–I** and Supplementary Figure S2, cells were incubated with 2.5% DSS for 48 h, and with IFN-γ (1000 U) or LPS (500 ng/ml) for 72 h respectively, a significant down-regulation of p-AMPK with decreased expression of tight junction proteins (i.e., ZO-1, claudin-1 and occludin) was observed by western blot. However, protein level of p-AMPK, tight junction proteins was markedly increased in the presence of metformin (10, 30, and 100 μM).

**FIGURE 7:**
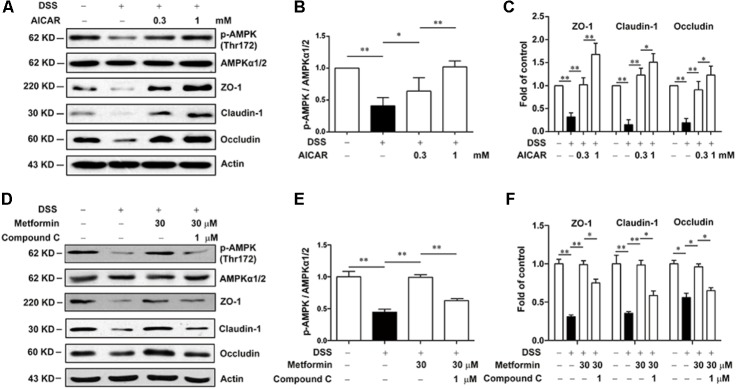
Metformin increased the expression of tight junctions in an AMPK-dependent way in Caco2 cells *in vitro.* **(A–C)** Caco2 cells were treated with 2.5% DSS in the presence or absence of AICAR (0.3 and 1 mM) for 48 h. The expression of p-AMPK, AMPKα1/2, ZO-1, claudin-1 and occludin were determined by western blot. **(D–F)** Caco2 cells were incubated with 2.5% DSS and 30 μM metformin with or without 1 μM compound C, the expression of p-AMPK, AMPKα1/2, ZO-1, claudin-1 and occludin were determined by western blot. Quantifications of immunoreactive signals were performed after normalization to total protein content of each lane. Data are presented as mean ± SEM; ^∗^*P* < 0.05, ^∗∗^*P* < 0.01.

Next, we used Compound C and AICAR to test whether the effect of metformin was dependent on AMPK. As shown in **Figures 7A–C**, AICAR (0.3, 1 mM) significantly up-regulated the decreased expression of tight junction proteins induced by 2.5% DSS in Caco2 cells, which exhibited a similar effect of metformin. Nevertheless, AMPK phosphorylation caused by metformin was markedly inhibited by compound C (1 μM) in Caco2 cells, with a consequent reverse on the increased expression of ZO-1, claudin-1 and occludin mediated by metformin (**Figures 7D–F**).

### Metformin Promoted Assembly of Tight Junctions in Caco2 Cells *in Vitro*

**FIGURE 8:**
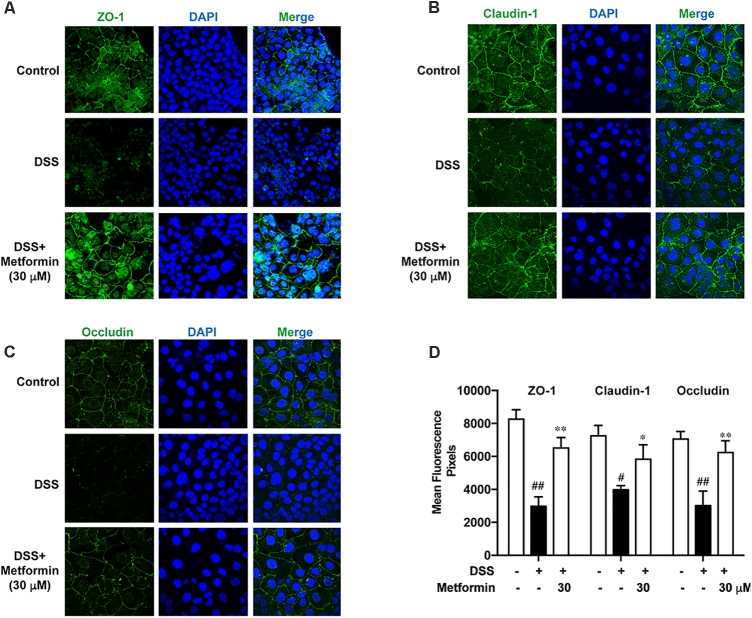
Metformin promoted assembly of tight junctions in Caco2 cells *in vitro*. **(A–C)** Immunofluorescence staining of ZO-1, claudin-1 and occludin. Caco2 cells were grown to confluence with or without 30 μM metformin and subjected to 2.5% DSS treatment for 48 h. **(D)** Quantification for ZO-1, claudin-1 and occludin fluorescence respectively. Magnification: 600×. ^#^*P* < 0.05, ^##^*P* < 0.01 *vs.* normal group, ^∗^*P* < 0.05, ^∗∗^*P* < 0.01 *vs.* DSS-treated alone group.

It is shown in previous studies that AMPK facilitates the assembly of tight junctions in epithelial cells (Calder et al., 2017; Prasad et al., 2017; Rowart et al., 2017; Wang et al., 2017). Here we observed the distribution of ZO-1, claudin-1 and occludin in the presence or absence of metformin (30 μM) in Caco2 cells after 2.5% DSS treatment for 48 h with immunofluorescence staining and confocal microscopy. As shown in **Figures 8A–D**, DSS treatment induced irregular morphology and distribution of ZO-1, claudin-1 and occluding. However, the membrane-distribution and assembly of these tight junction proteins were significantly enhanced by metformin. These data demonstrated that AMPK activation by metformin maintained the structure of tight junctions in colonic epithelial cells and increased their resistance to DSS damage.

## Discussion

In this study, we found that (Burkle and Hanfling, 2015) the active form of AMPK (phosphorylation on Tyr172) was decreased during the progression of colitis in colonic epithelial cells in mice; (Burkewitz et al., 2014) administration of metformin, a classic drug to activate AMPK, attenuated symptoms of colitis significantly; (Zheng and Cantley, 2007) the underlying mechanism of preventing colitis by metformin relied on its regulation of tight junctions via AMPK activation. Collectively, these results illustrated the relevance between colonic epithelium damage and AMPK function. They may provide a beneficial strategy for treating colitis using an AMPK-activator clinically.

AMPK, a serine/threonine kinase, is a highly conserved energy sensor. It is activated by phosphorylation on Thr172 in the catalytic subunit (α) by LKB1, CAMKK2, ATP-to-AMP ratio and so on (Herzig and Shaw, 2017). Under conditions of low energy, activated AMPK phosphorylates downstream substrates, leading to ATP generation and decreases ATP consumption, thus modulating energy metabolism (Hutber et al., 1997; Hardie, 2007). Accumulating evidence has proved that there are marked dysfunctions in metabolic regulation in immune cells in many diseases, including inflammatory conditions. For example, activated macrophages and T-helper 17 cell have increased glucose uptake and glycolysis; conversely, anti-inflammatory cells, such as M2 macrophages, regulatory T cells, have lower glycolytic rates and higher levels of oxidative metabolism. AMPK activation creates a pseudo-starving state that can promote oxidative metabolism and inhibits inflammation through many signaling networks, such as nuclear factor κB and mitogen-activated protein kinases (MAPK) signaling pathway (O’Neill and Hardie, 2013; Song et al., 2015; Peixoto et al., 2017; Di Fusco et al., 2018). Hence, AMPK is a crucial regulator of these metabolic dysfunction responses in immune cells.

However, during the progression of colitis, epithelial cells also play important roles in keeping integrity of intestinal barrier and the role of epithelial AMPK remains elusive. Although a recent study has revealed that AMPK deletion exacerbates DSS-induced colitis and AMPK could promote gut epithelial differentiation and barrier function (Sun et al., 2017), the changes of AMPK during colitis is still unclear. In the present study, we found that during the initiation and development of colitis, AMPK phosphorylation on Thr172, as the activate form of AMPK, was gradually decreased while the expression of total AMPK was unchanged, and reactivation of AMPK by metformin could significantly reduce the severity of DSS-induced colitis. Since AMPK is not the sole target for metformin, we used Compound C, a specific inhibitor of AMPK, to examine whether the effect of metformin on colitis and the integrity of tight junction is dependent on AMPK activation. As shown in **Figures 5, 7D–F**, Compound C could reverse the effect of metformin in both *in vivo* and *in vitro* experiments. Moreover, AICAR, another AMPK activator exhibited a similar effect on tight junction as metformin (**Figures 5, 7A–C**). These results indicated that the effect of metformin on colitis and tight junction was dependent on AMPK activation.

During the pathological process of IBD, the impaired intestinal barrier function can increase permeability of intestinal tract, triggering pathogen bacteria into lamina propria (Jenkins et al., 1987; Buhner et al., 2006). The harmful production like LPS, released by pathogen bacteria could induce immunological disorders, such as infiltrating and activating macrophage, inducing excessive effector T cells and recruiting neutrophils, resulting in overproduction of many pro-inflammatory cytokines (i.e., IFN-γ, IL-1β, IL-18, IL-6), which exacerbate colitis to a great extent(Bouma and Strober, 2003; Sartor, 2006). Hence, in addition to DSS, we also used IFN-γ or LPS to mimic the environment of colitis in Caco2 cells respectively (Araki et al., 2006; Cao et al., 2013; Satyam et al., 2017), and we found that metformin could up-regulate the decreased expression of tight junction proteins upon these three stimulations (**Figure 6**).

It is worth noting that, the effects of metformin on cytokine profiles are not on dose dependent manner (**Figure 3C**), which seems contradictory with the therapeutic effect of metformin in colitis. As is widely accepted, colitis is a kind of complex disease, including many pathological processes, such as intestinal barrier dysfunction and inflammatory disorders, and they may play different roles in the different time points during the progression of colitis. We speculated that during different pathological processes, metformin functions via different mechanisms. For example, many signaling pathways are involved in inflammatory responses in immune cells, and in addition to AMPK, metformin could target to some other crucial points directly (Buldak et al., 2014; Koh et al., 2014; Pandey et al., 2017), so the dosage of metformin towards inflammation seems to be lower than that towards epithelial tissue. In terms of intestinal epithelial cells, amounting studies have proved that AMPK plays an important role in regulation of tight junctions. These effects have been mechanically implicated in some previous works that AMPK, depending on the kinase activity of LKB1, might promote ZO-1 assembly in MDCK cells (Zhang et al., 2006; Zheng and Cantley, 2007); and AMPK can promote gut epithelial differentiation and barrier function through accelerating the expression of the intestinal caudal type homeobox 2 (CDX2), a key transcription factor for intestinal differentiation via histone modifications (Silberg et al., 2000; Sun et al., 2017). Collectively, AMPK is the pivotal target for metformin in intestinal epithelial cells. Hence, the dose of metformin on regulation of tight junction was higher than the dose on anti-inflammatory in immune cells. However, the different effect of metformin on inflammatory or tight junction – remains to be intensively studied in future.

To date, metformin has been demonstrated to have varieties of effects other than anti-diabetes. Its potential clinical applications continue to be expanded, such as anti-tumor, anti-inflammation and anti-aging effects. Although the mechanisms of these pharmacological effects still remain to be clearly elucidated, it is widely accepted that AMPK is the most important target of metformin. It can activate AMPK by inhibiting mitochondrial complex I in respiratory chain to increase the AMP/ATP ratio (Owen et al., 2000). In this study, we reported for the first time that in addition to its anti-inflammation effect, metformin can also improve the mucosal integrity via promoting expression and assembly of tight junctions in an AMPK-dependent way. More importantly, metformin exerts a greater effect than mesalazine on attenuation of colitis induced by DSS, which makes metformin more attractive in IBD treatment. Given to the safety and effectiveness of metformin, there is an enticing prospect of new therapeutic approaches for inflammatory diseases.

## Author Contributions

XfW and QX designed research. LC, JW, QY, SH, QM and JG performed experiments. LC and QY analyzed data. XdW, YSh, and YSu supported materials and gave suggestions in the formation and revision of the manuscript. LC and XfW wrote the manuscript.

## Conflict of Interest Statement

The authors declare that the research was conducted in the absence of any commercial or financial relationships that could be construed as a potential conflict of interest.
