# Supplementary material for: Activating AMPK to Restore Tight Junction Assembly in Intestinal Epithelium and to Attenuate Experimental Colitis by Metformin
Source: Front Pharmacol. 2018 Jul 16;9:761. doi: 10.3389/fphar.2018.00761 (PMC6054982; doi:10.3389/fphar.2018.00761)

**Supplymentary data**

**Figure 1** IgG for rabbit and mouse was stained by immunohistochemistry experiments.

**
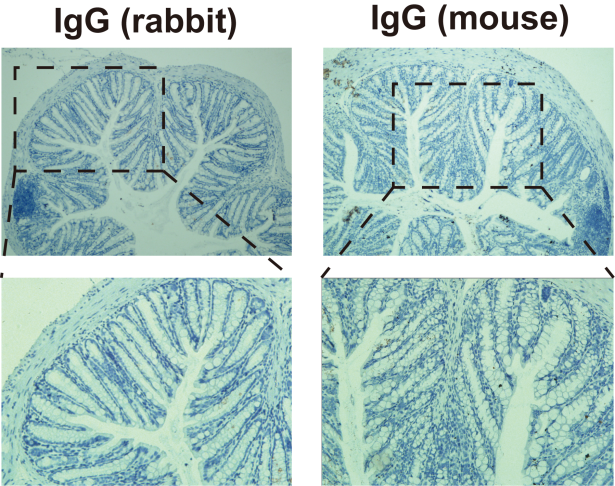
**

**Figure 2** MTT assay was performed to examine the viability of Caco2 cells co-cultured with different concentrations of metformin.


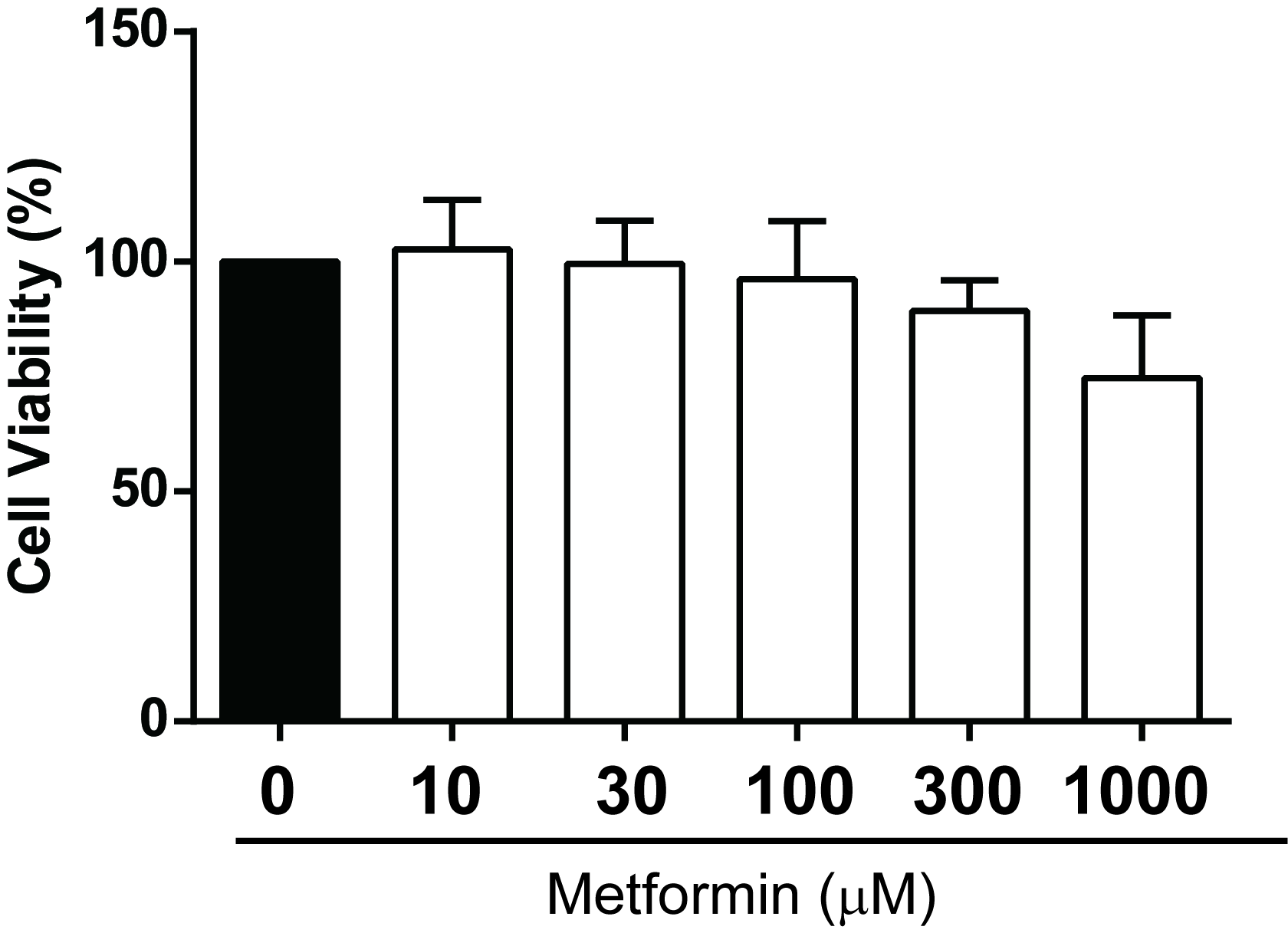

Supplement: Supplementary file 1 [file Table_1.DOCX]
